# Meteorological Normalisation Using Boosted Regression Trees to Estimate the Impact of COVID-19 Restrictions on Air Quality Levels

**DOI:** 10.3390/ijerph182413347

**Published:** 2021-12-18

**Authors:** Sandra Ceballos-Santos, Jaime González-Pardo, David C. Carslaw, Ana Santurtún, Miguel Santibáñez, Ignacio Fernández-Olmo

**Affiliations:** 1 Department of Chemical and Biomolecular Engineering, University of Cantabria, 39005 Santander, Spain; jaime.diez@unican.es (J.G.-P.); ignacio.fernandez@unican.es (I.F.-O.); 2 Wolfson Atmospheric Chemistry Laboratories, University of York, York YO10 5DD, UK; david.carslaw@york.ac.uk; 3 Ricardo Energy & Environment, Didcot OX11 0QR, UK; 4 Unit of Legal Medicine, Department of Physiology and Pharmacology, University of Cantabria, 39011 Santander, Spain; ana.santurtun@unican.es; 5 Global Health Research Group, Department of Nursing, University of Cantabria, 39008 Santander, Spain; miguel.santibanez@unican.es; 6 Research Nursing Group, IDIVAL, Calle Cardenal Herrera Oria s/n, 39011 Santander, Spain

**Keywords:** air pollution, COVID-19, lockdown, deweather, meteorological normalisation, boosted regression trees

## Abstract

The global COVID-19 pandemic that began in late December 2019 led to unprecedented lockdowns worldwide, providing a unique opportunity to investigate in detail the impacts of restricted anthropogenic emissions on air quality. A wide range of strategies and approaches exist to achieve this. In this paper, we use the “deweather” R package, based on Boosted Regression Tree (BRT) models, first to remove the influences of meteorology and emission trend patterns from NO, NO_2_, PM_10_ and O_3_ data series, and then to calculate the relative changes in air pollutant levels in 2020 with respect to the previous seven years (2013–2019). Data from a northern Spanish region, Cantabria, with all types of monitoring stations (traffic, urban background, industrial and rural) were used, dividing the calendar year into eight periods according to the intensity of government restrictions. The results showed mean reductions in the lockdown period above −50% for NO_x_, around −10% for PM_10_ and below −5% for O_3_. Small differences were found between the relative changes obtained from normalised data with respect to those from observations. These results highlight the importance of developing an integrated policy to reduce anthropogenic emissions and the need to move towards sustainable mobility to ensure safer air quality levels, as pre-existing concentrations in some cases exceed the safe threshold.

## 1. Introduction

Coronavirus (COVID-19) disease, caused by Severe Acute Respiratory Syndrome Coronavirus 2 (SARS-CoV-2), was first detected in Wuhan (China), in late December 2019, and spread rapidly across the globe in the following months, leading to an unprecedented public health and economic crisis in the 21st century [1] that will surely have huge long-term social and economic impacts [2]. The World Health Organization (WHO) declared COVID-19 a “global pandemic” on 11 March 2020 [3]. To control the disease outbreak, governments established very restrictive containment measures, such as social distancing, quarantines and isolation. 

The first case of COVID-19 positive in Spain was identified on 30 January 2020, and the number of infections increased exponentially, resulting in one of the highest mortality rates [4]. The Spanish authorities declared the State of Alarm and strict lockdown on 14 March 2020 [5]. This lockdown was even tighter during the period 30 March to 9 April, when non-essential activities were totally prohibited [6], forced by the overburdened intensive care units and health systems [7]. After one month of lockdown to flatten the epidemic curve, the situation seemed to be under control [6]. Thus, since 1 May 2020, the restrictions have been progressively and asymmetrically relaxed depending on the pandemic indicators of each region, in accordance with a three-phase de-escalation plan aimed at enabling a return to the “new normality” [8]. By 21 June, the State of Alarm ended along with most limitations in most of the country. During the summer months, positive cases were minimal [9], but the situation worsened at the end of October with a second large wave of infections. This led to the declaration of a Second State of Alarm on 25 October 2020 [10] that lasted until 9 May 2021. 

Despite the devastating effects of the pandemic, the environment benefited in multiple ways, at least during lockdown [11]. Improvements in air quality levels across all scales, from regional to global, resulted from the reduction of anthropogenic emissions because of severe mobility restrictions, reduced industrial activity and other measures taken to combat the spread of the virus [12]. This situation provides a unique opportunity to investigate in detail the impacts of these restricted anthropogenic emissions on air quality [13], and to better understand the complex response of the atmosphere to human activities [14,15], which offers insights into the prioritisation of future clean air actions [16]. This issue awakened the interest of the general public (due to the known effects of air pollution on climate change and on human health), the authorities and, in particular, the scientific community, giving rise to a “boom” of studies worldwide reporting significant reductions in the levels of the main air pollutants and some increases in tropospheric ozone concentrations in urban environments [17,18].

There are a wide range of strategies and approaches to investigate the impact of the COVID-19 crisis on the air quality. Some authors have used satellite images from the Copernicus Sentinel-5P Tropospheric Monitoring Instrument (TROPOMI) to display substantial reductions in NO_2_ levels over multiple cities across Europe, Asia and America [6,11,13,19,20,21,22,23]. However, although satellite measurements show a rapid, global picture of concentrations over large areas, they may not provide sufficient resolution for local effects and may bias the data when comparing different regions, as satellites overpass them at different local times [12]. Ground-based measurements have also been commonly used; in a recent review carried out by Gkatzelis et al. [18], they comprised the largest fraction of the data used in the analysis of COVID-19 lockdowns. These data usually come from local, regional or national air quality monitoring networks, which provide preliminary data in near real time, with a final quality-assured data update process. Given the speed with which these publications were prepared, it is possible that many are based on inhomogeneous data, for a very short period, and without final quality control, so the results may not be very reliable [24]. 

Once the air quality database is complete, different methodologies can be used to compare observed concentrations to a “business as usual” (BAU) reference period [25], in which emissions are maintained as if no extraordinary event had occurred [26]. For this purpose, two main approaches can be distinguished: (a) a comparison of pollutant concentrations directly before and/or after lockdown [14,27,28] or (b) a comparison of pollutant concentrations from seasonally similar time periods, including this same period of 2019 [29,30], and often several years before [12,20,31]. The former often covers relatively short time periods, with uncertainties associated with the unaccounted effects of seasonality and meteorology. Although the latter partially considers these effects, uncertainties may still arise from the meteorological variability and exceptional events [18]. 

Therefore, another key aspect that can determine the quality of the study is accounting for meteorological effects [32]. Many studies, especially the first published ones, did not take into account the weather influence, and it is well known that it plays a crucial role in the formation, transport, deposition and transformation of air pollutants [33,34]. Thus, unfavourable meteorological conditions can lead to days of heavy pollution, even if the total emission is reduced [35,36,37,38]. If meteorology is not controlled for, the fluctuations observed in pollutant concentrations may be masked by meteorological variation rather than emission reductions [39], which can lead to wrong conclusions regarding the assessment of the environmental impact of an unusual event [40,41]. In addition to this, when comparing pollutant concentrations with those of previous years, it is vital to take into account trends in emission patterns [16,42], especially for those pollutants that have shown important reductions over time due to regulations or other legislative restrictions [43,44,45]. Consequently, it is essential to decouple these effects from ambient air quality data, while quantifying the real changes in atmospheric pollution due to COVID-19 restrictions [46].

Meteorological normalisation is a technique that can be used to control for meteorology over time in air quality time series by reducing variability using statistical modelling [47]. Variability reduction is achieved by training a model that can explain part of the variation of pollutant concentrations through a number of independent variables [48]. The variables commonly used are surface-based meteorological observations (wind speed and direction, temperature, solar radiation, precipitation, etc.) and temporal variables such as hour of day and season [43,45]. Once the model has been trained and tested, concluding that it can describe a sufficient part of fluctuations, it can be used to remove the confounding influences that the independent variables have on the dependent variable by sampling and predicting [16]. The normalised time series are in the original units of the pollutant and can be considered as “average” concentrations or levels at meteorological fixed conditions [49]. However, the prediction of air pollutant concentrations is non-trivial and challenging, especially with parametric methods, due to the complexity of the mechanisms involved, which are often non-linear [50,51].

More recently, new methodologies based on regression decision trees have been developed, which are suitable for weather normalisation [52]. These include the Boosted Regression Trees (BRT) and Random Forest (RF) algorithms [43,50,53,54]. These machine learning-based techniques perform better than traditional statistical and air quality models by improving some performance metrics, such as variance and bias, in high-dimensional datasets [45]. BRTs offer many advantages over competing techniques for prediction, which are highly relevant to air quality analysis. They are able to handle a mix of variables, including continuous and categorical variables, which do not need to be transformed for fitting [40]. Moreover, missing data can be managed efficiently, trees can model non-linear effects and interactions between variables, and they are resistant to outliers in the variable space [51]. Boosted Regression Trees were previously employed to analyse air pollution data at mixed source locations [43] and to quantify the short-term impact of interventions [40]. However, to the best of our knowledge, no studies have been published using R packages that integrate BRT models, similar to RF-based models such as “rmweather” or “normalweatherr”, which have been recently used to study the impact of COVID-19 on air pollution [16,49,55].

The aim of this investigation was to apply a weather and emission trend normalisation procedure to estimate the impact of COVID-19 restrictions on air quality levels over the full year 2020. The analysis of the entire year allowed accounting for the different intensities of the restrictions by comparing the periods allocated in 2020 (pre-lockdown, lockdown, etc.) with the same periods of a reference dataset (2013–2019). The normalisation strategy was based on BRT models implemented in “deweather”, an R package previously developed by Carslaw [56] (https://github.com/davidcarslaw/deweather, accessed on 20 March 2021), comparing the 2020 normalised results with the previous seven-year normalised average to account for long-term trends. To apply this methodology, a dataset from a small region (Cantabria, Northern Spain) with all type of stations (traffic, industrial, urban background and rural) was used to investigate whether the impacts derived from COVID-19 restrictions can be quantified in a small regional air quality network.

## 2. Materials and Methods

### 2.1. Area and Period of Study

**Figure 1:**
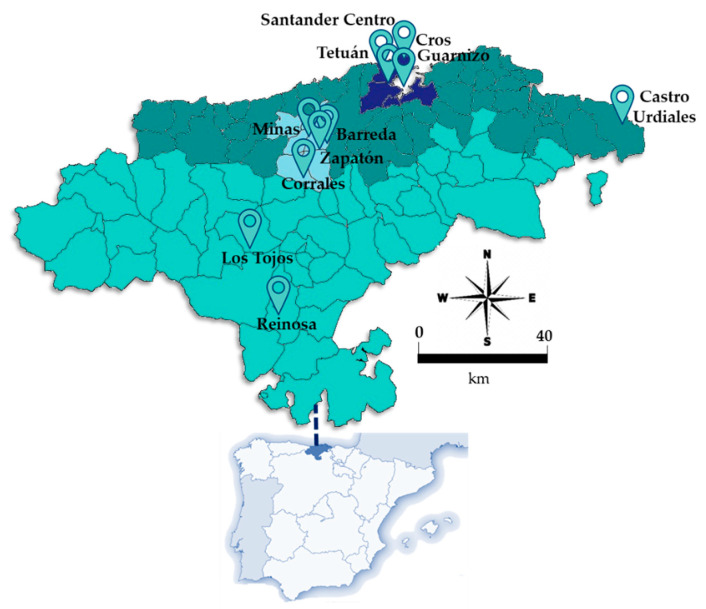
Locations of the 11 air quality monitoring stations within the study region of Cantabria, (Northern Spain). Colours denote the four zones considered in this region: Santander bay (dark blue), coastal zone (dark green), Torrelavega area (light blue), and inner zone (light green).

The study focused on the region of Cantabria, in Northern Spain. The spatial distribution of the population living in this region, constituted by 102 municipalities, is not homogeneous, with 30% of the total inhabitants residing in the city of Santander [57]. The industrial activity is mainly concentrated in two areas: Santander and Torrelavega. Therefore, these cities usually have higher air pollution levels [58]. Despite being a small region (5321 km^2^), the air quality-monitoring network of Cantabria is formed by 11 stations (Figure 1) of all types: traffic, urban background, industrial and rural. This offered the chance to evaluate the impact of COVID-19 restrictions across all the environments.

**Figure 2:**
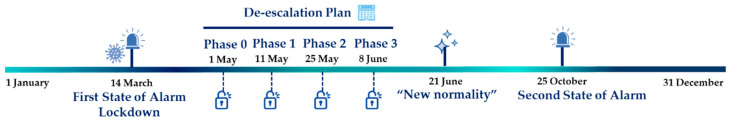
Chronology of the COVID-19 periods in Cantabria (year 2020).

To estimate the effect of the crisis during the lockdown, and the subsequent stages, the study covered the full year 2020, divided as follows (Figure 2): (i) Pre-lockdown (1 January–13 March), (ii) Lockdown (First State of alarm) (14 March–30 April), (iii) Phase 0 (1–10 May), (iv) Phase 1 (11–24 May), (v) Phase 2 (25 May–7 June), (vi) Phase 3 (8–20 June), (vii) “New normality” (21 June–24 October), and finally (viii) Second State of Alarm (25 October–31 December).

### 2.2. Air Quality and Meteorological Data

Air quality data for four key pollutants (NO, NO_2_, PM_10_ and O_3_) with 1 h resolution from 2013 to 2020 were retrieved from the European Air Quality Portal [59] using the “saqgetr” R package [60,61], freely available from the CRAN repository (https://cran.r-project.org/, accessed on 1 February 2021). In addition, to check for errors and missing periods of data, pollutant concentration data were also collected from the regional Air Quality Monitoring Network, managed by the Environmental Research Centre of Cantabria [62], which is integrated by 11 fixed stations throughout the territory, as Figure 2 illustrates. Table 1 shows the names, identification codes and classification of the sites into traffic, industrial, urban background and rural; the pollutants monitored at each site are also indicated. A database composed of all station types was employed to study the impact of restrictions in all environments and to draw more specific conclusions that may be useful for the development of future air policies. The selection of these pollutants was based on those with the highest levels, closest to the regulations. Missing data did not represent more than 5% at any site for the total span and were therefore ignored, as they would not affect the overall results due to the huge amount of data used in this study.

Meteorological data were obtained from surface measurements at the air quality monitoring stations specified in Table 1. For the remaining sites that did not cover these parameters, meteorological data were downloaded from the National Oceanic and Atmospheric Administration’s (NOAA) global Integrated Surface Database (ISD) using the R package “worldmet” [63], which is also freely available from the CRAN repository. From this source, data were acquired from the Spanish Meteorological Agency station located at Santander–Parayas airport [64]. An hourly meteorological database including wind speed (ws) [m/s], wind direction (wd) [°], temperature (air-temp) [°C], relative humidity (RH) [%], solar radiation (SR) [W/m^2^], cloud cover (cl) [oktas], and precipitation (LL) [L/m^2^] was completed with a 90% or above data capture on each parameter, although not all the variables were available at all the sites. The selection of these variables was based on those most closely related to significant changes in pollutant concentrations [43]. Besides, previous work that aimed to predict hourly concentrations of pollutants showed that models based on more readily accessible meteorological surface measurements were favourable relative to those that used more sophisticated input data (e.g., atmospheric stability and planetary boundary layer), and produced results with higher explanatory power [40].

### 2.3. Model Development

The weather normalisation procedure to remove meteorological variation from air quality data was conducted using the “deweather” R package [56]. It is part of the “Openair” suite of packages designed to carry out air quality and related data analysis [65]. The fundamentals of the “deweather” package are as follows: it uses a two-step procedure to normalise air quality data. First, models based on the BRT approach for daily concentration of selected air pollutants from different predictors are fitted using the “gbm” package [66]. Then, a meteorological averaging procedure is applied by predicting many times with a random sampling of weather conditions [43,56], using the “metSim” function.

BRTs combine regression trees (i.e., models that relate a response to its predictors) and boosting, an iterative method for developing a final model, progressively adding trees, while re-weighting data to emphasise cases poorly predicted by previous trees [67]. Regression trees model the independent variable *Y* by means of stratifying or segmenting the predictor space *X* into a number of simple regions. This involves recursive binary splits of each predictor *X*_j_ (different predictors *X*_1,_ *…, X*_p_ can be considered), at the cutpoint *s* such that splitting the predictor space into the regions R_1_ (*j,s*) = {*X|X*_j_ < s} and R_2_ (*j,s*) = {*X|X*_j_ ≥ s} leads to the greatest possible reduction in the residual sum of squares, minimizing Equation (1):

(1) $$\underset{i:\;x_{i}\in R_{1}\left(j,s\right)}{\sum }{\left(y_{i}-{\hat{y}}_{R_{1}}\right)}^{2}+\underset{i:\;x_{i}\in R_{2}\left(j,s\right)}{\sum }{\left(y_{i}-{\hat{y}}_{R_{2}}\right)}^{2}$$

where ${\hat{y}}_{R_{1}}$ is the mean response for the training observation in R_1_ (*j,s*), and ${\hat{y}}_{R_{2}}$ is the mean response for the training observations in R_2_ (*j,s*).

The final boosted model $\hat{f}$ is obtained as a combination of a large number of regression trees *f^b^* (Equation (2)):

(2) $$\hat{f}\left(x\right)=\overset{ntree}{\underset{b=1}{\sum }}\lambda {\hat{f}}^{b}\left(x\right)$$

where λ is the shrinkage or learning rate parameter. Each regression tree *f^b^* is fitted with d splits (interaction depth) to the training data (*X*, *r_i_*) updating the residuals *r_i_* on each iteration (Equation (3)) [68]:

(3) $$r_{i}\leftarrow r_{i}-\lambda {\hat{f}}^{b}\left(x_{i}\right)$$

In more detail, statistical models were developed using the “gbm” package to explain concentrations based on the meteorological variables cited in the previous section, and temporal variables to forecast the variability linked to the hour of the day, the day of the week and the week of the year. The latter accounted for seasonal meteorological effects not considered by the other parameters. Moreover, the algorithm included a trend term to capture longer-term changes in emission patterns over the period of seven years considered. For each site, the hourly meteorological and pollutant measurements recorded across the total period were randomly divided (by default) into a fraction to train the BRT models (80%) and a fraction for testing its performance (20%), with the aim of developing the most appropriate model. This decision is made automatically with the evaluation of common metrics such as Pearson´s correlation coefficient (*r*), root mean square error (RMSE) and mean bias (MB). In this study, individual models were built for each pollutant and period of analysis, i.e., 2020 and the reference period (2013–2019). The model fitting parameters were kept as defaults (learning rate = 0.1 and interaction depth = 6.0) as these values were previously optimised using 10-fold cross validation (CV) by other authors [43]. The interaction depth accounts for the interaction between variables. Different number of trees (n.trees) were tested and finally a value of 1000 was used as a compromise between computational time and model performance.

#### 2.3.1. Meteorological Normalisation

**Figure 3:**
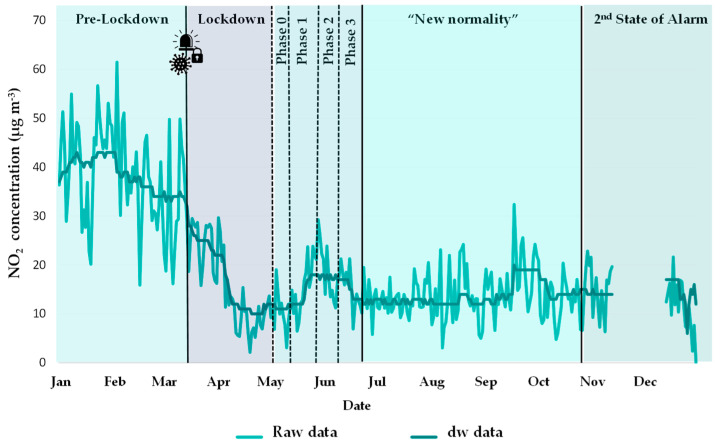
Removal of meteorological variability from the raw data of 2020 for NO_2_ at the Santander Centro site using the “deweather” package.

Once the model was built, the meteorological averaging procedure was applied by predicting many times with random sampling of weather conditions [56]. This sampling was carried out by the “metSim” function. It is important to note that the model was not used to predict the counterfactual or business as usual (BAU) 2020 scenario, but to predict 2020 concentrations taking into account the drop caused by COVID-19, and removing the meteorological variability. In practice, new time series of concentrations are generated for random samples of meteorology hundreds of times. This approach yields a single, new time series of predicted concentrations that represents average meteorology. An example of the deweathered (dw) time series for 2020 is shown in Figure 3.

#### 2.3.2. Emission Pattern Trends Normalisation

**Figure 4:**
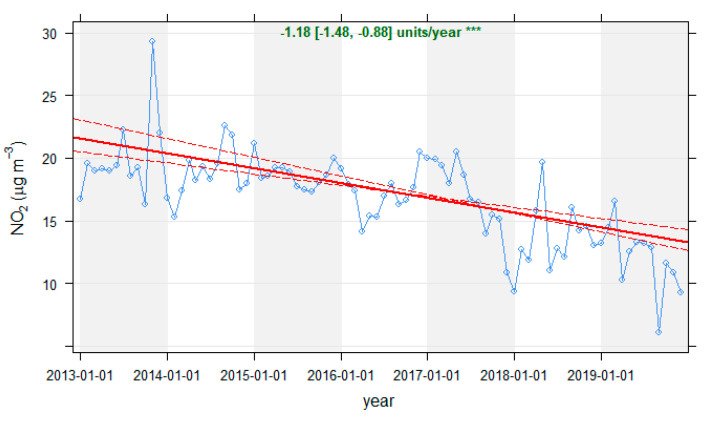
Trend analysis for NO_2_ concentrations from 2013 to 2019 at the Zapatón site, using the “TheilSen” function. Overlaid is shown the slope with the 95% confidence intervals (*p*-value < 0.005).

**Figure 5:**
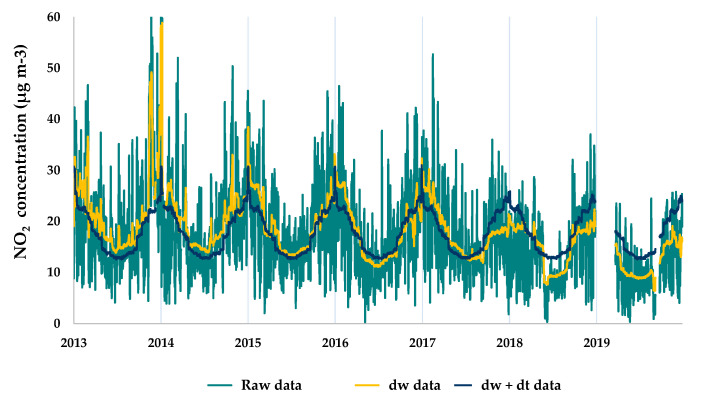
Trend normalisation of NO_2_ concentrations from 2013 to 2019 at the Zapatón site, using the “deweather” package [10].

Data concentrations of the reference period (2013–2019) were analysed using the “TheilSen” function implemented in “Openair”, to characterise general air quality trends prior to lockdown. The method provided a non-parametric measurement of trends on “a median of slopes of pairs of points with different x-values” estimation of the slope (Figure 4), and bootstrap estimation of uncertainty [69]. These trends from previous years may mask the results, leading to erroneous conclusions, so a similar procedure to weather normalisation was employed to remove them. By including the “trend” term in the “metSim” function, emission trends were averaged, so the reference period converted into a fixed emissions scenario [43]. An example of the detrended (dt) time series is shown in Figure 5.

It is important to highlight that, because the study made a daily comparison, i.e., each day of the year 2020 was compared with the same day of the average (2013–2019), the variable “week” was not normalised, as this variable represented the seasonal changes attributed to each week of the year, which were observed in both data series. By contrast, the day of the week was normalised, because weekly patterns of pollutant concentrations are important, mainly due to differences between working days and weekends [70].

### 2.4. Quantification of Changes

Equation (4) was applied to raw (unnormalised) data to calculate the observed changes in air pollutant levels. The percentage of change (*P*) in each period (*i*) was obtained individually for each air pollutant at each site as follows:

(4) $$P\left(\%\right)\;period\;i=\frac{\bar{C_{2020,period\;i}}-\bar{C_{2013-2019,\;period\;i}}}{\bar{C_{2020,period\;i}}}\;\times 100$$

where $\bar{C_{2020,period\;i}}$ corresponds to the average concentration of a given pollutant at a given station, in each period *i* of the year 2020: pre-lockdown, lockdown, phase 0, phase 1, phase 2, phase 3, “new normality” and 2nd State of Alarm; and $\bar{C_{2013-2019,period\;i}}$ represents the mean concentration of the same pollutant in the same period, averaged over the last seven years.

In order to further obtain the normalised percentage changes (*P_dwdt_*) (after removing meteorological and emission trend influences), a similar process using Equation (5) was followed:

(5) $$P_{dwdt}\left(\%\right)\;period\;i=\frac{\bar{C_{\left(2020\right)dw,period\;i}}-\bar{C_{\left(2013-2019\right)dw+dt,\;period\;i}}}{\bar{C_{\left(2020\right)dw,period\;i}}}\;\times 100$$

As explained in Section 2.3, the “deweather” procedure was applied to the complete database, whereas the “detrend” procedure was only implemented for (2013–2019) data; thus, the terms in Equation (2) were $\bar{C_{\left(2020\right)dw,period\;i}}$ and $\bar{C_{\left(2013-2019\right)dw+dt,period\;i}}$, respectively.

## 3. Results and Discussion

### 3.1. Observed Changes

**Figure 6:**
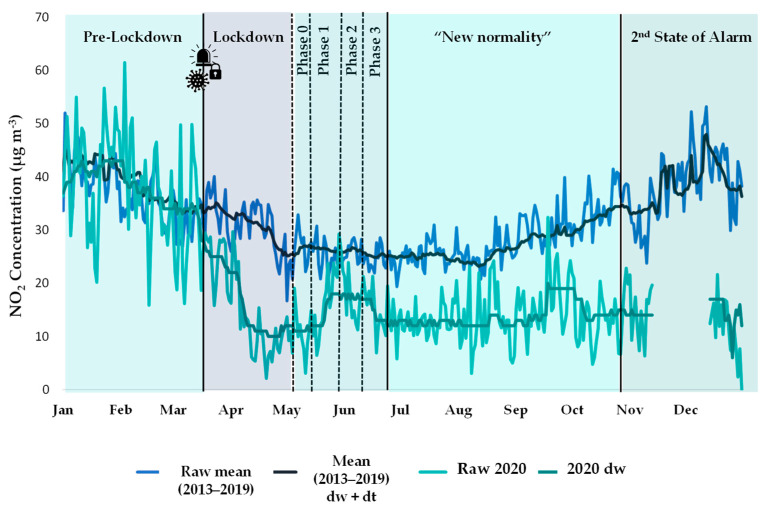
Observed (raw) and normalised daily NO_2_ concentrations at the Santander Centro site over a calendar year: comparison between 2020_dw_ data and the average of the previous seven years (2013–2019)_dw + dt_, after applying the procedure to remove meteorological and emission trend influences.

**Figure 7:**
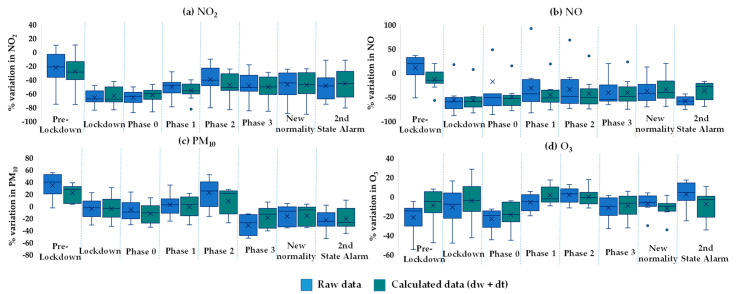
Box plots of variations (%) in pollutant concentrations (**a**) NO_2_, (**b**) NO, (**c**) PM_10_, (**d**) O_3_, at all studied sites from raw data (blue) and calculated data (green) after using the “deweather” package, comparing 2020 levels with the mean of the previous seven years (2013–2019) in each period. Note that arithmetic mean values are marked with a cross and whiskers represent the minimum and maximum non-outlier values.

Figure 6 (light blue and green series) presents the observed daily NO_2_ concentrations for the full year 2020 at Santander Centro (except for a short period in December when no data were available) divided into periods according to COVID-19 restrictions and compared with the mean levels recorded in the previous seven years. During the lockdown, concentrations registered a significant drop. In the following periods of de-escalation, with the relaxation of some limitations, levels increased but remained far below the previous years. Finally, during the Second State of Alarm, which was much less stringent than the first in terms of mobility, NO_2_ concentrations remained more than halved at this location. This comparison was obtained for each pollutant at each site, but to condense the results, they were grouped and summarised in a box plot (Figure 7, series in blue). Nevertheless, in some cases the pollutant concentrations at a specific site, such as PM_10_ at Los Tojos, are not included in the chart because of some anomalies that would have distorted the results.

Figure 7 depicts that mean pre-lockdown levels were lower than the baseline period for pollutants such as NO_2_ and O_3_, whose concentrations in 2020 were around −23% in both cases, whereas for NO and PM_10_ the year started with higher levels, +10% and +34%, respectively. These changes varied greatly between sites, suggesting that different emission and meteorological patterns occurred in early 2020 (i.e., before the lockdown) with respect to the reference period. Querol et al. [71] analysed the meteorological patterns during the pre-pandemic period in Spain, comparing it with the previous years (2015–2019), and found weather anomalies that induced higher than usual wind speeds, temperature increases, lower precipitation, and cloudiness over the Bay of Biscay, which is close to the region studied here. Under that scenario, they reported gains in PM_10_ levels in pre-lockdown in Bilbao that ranged from +1% (at urban background sites) to +23% (at traffic sites). The cited values were obtained after the subtraction of African dust outbreaks which were abundant in the north of the peninsula during the pre-lockdown and the early days of the lockdown, as reported by the Ministry for the Ecological Transition and the Demographic challenge [72]; thus, they seem to be in line with our findings (from raw data without removing dust intrusions). They also quantified an average reduction of −22% in NO_2_ and −8% in O_3_, the latter being significantly lower than our results, although the comparison is not straightforward as they used 8 h measurements whereas we used daily averaged concentrations.

The lockdown of 14 March led to significant decreases in nitrogen oxide concentrations, −65% for NO_2_ and −57% for NO (average of the sites considered) (see Figure 7), as a result of a drastic reduction in traffic, of up to −90% in some Spanish cities [73]. These results are comparable to the observations of Tobías et al. [6] and Baldasano [74] for traffic sites in Barcelona (−51%) and in Madrid (−62%) [18]; Querol et al. [71] published, as an average, −51% for eleven cities in Spain considering several site types; and Ordóñez et al. [75] observed at urban sites a decrease of −51.6% in Spain. These findings also confirmed the general trend found worldwide by the experts [11,19,33,75,76,77,78,79], including Spain among the three most affected European countries with the highest NO_2_ reductions. However, the measured average reduction in PM_10_ was below −5% and O_3_ decreased by −10%. These percentages express the variation with respect to the baseline (2013–2019), but considering the changes in lockdown versus the pre-lockdown period, the outcomes are somewhat different for some pollutants.

Taking average PM_10_ reductions as an example, when comparing both periods (i.e., before and during lockdown) it was observed that the change went from a positive (+34%) to a negative gain (−5%). Thus, the decrease was substantial, but perhaps not directly induced by the COVID-19 restrictions because the variation with respect to the baseline scenario was minimal. This highlights the relevance of choosing a robust comparison period. Many papers showed large reductions in air pollution levels, but if they simply compare the levels in lockdown with the previous months, they do not account for seasonality, which is crucial when studying air pollution trends, as emphasised during the introduction. Globally, the impact of COVID-19 on PM_10_ levels ranged from −9 to −60%, according to a recent review carried out by Marinello et al. [17]. Querol et al. [71] obtained lockdown changes in PM_10_ that varied significantly from one Spanish city to another, and between the different site types studied. Lovrić et al. [49] also claimed reductions in PM_10_ that were not as pronounced as in NO_2_ in the Austrian city of Graz. As they explained, that fact demonstrated that traffic is just one of many contributing sources to PM_10_. Thus, some natural sources of PM_10_, such as marine aerosol, crustal material including Saharan intrusions, and some secondary inorganic aerosols not directly related to road traffic (ammonium sulphate) may notably contribute to the levels of PM_10_ measured in some of these studies, as reported in a former study carried out in the Cantabria region [80].

With respect to O_3_, it is necessary to point out that the observed variation during lockdown, −10% on average below the baseline at the region studied, did not follow the general trend recorded across Europe by authors such as Grange et al. [55], where mean O_3_ concentrations increased by a similar magnitude to the decrease in NO_2_. In fact, they reported an increase in O_3_ levels in Spain of between +37 and +61%, comparing the observed values with those estimated in the BAU scenario. This O_3_ increment linked to the lockdown was also found in Spain by Gorrochategui et al. [81] and Tobías et al. [6]. However, although Ordóñez et al. [75] reported O_3_ increases over most of Europe, both at urban background and rural sites, an average raw reduction of −10% was found in Spain.

However, it is necessary to highlight that important differences between sites were registered in the Cantabrian network, with positive relative changes in O_3_ concentrations at Tetuán (Santander urban background) and Cros (industrial) sites, indicating the importance of local sources of different precursors on O_3_ formation. In this line, Querol et al. [70] described different responses of urban O_3_ but a generalized and light decrease in rural locations. Therefore, this needs to be further investigated, bearing in mind the complex behaviour of O_3_ during the lockdown, which can be partially explained by the alteration of the photolytic cycle due to a strong decrease in NO levels in a VOCs-limited environment (usually urban areas) [82], which acts on the consumption of O_3_ in the titration reaction (NO + O_3_ ↔ NO_2_ + O_2_) [49,83]_._ However, the different intensities of reductions in emissions of other O_3_ precursors, such as NMVOCs, may have led to different levels of tropospheric O_3_ in different regions, according to the results found by other authors [84,85]. In addition, considerable O_3_ production downwind of sources only happens during periods of sustained insolation and high temperatures. Hence, the role of meteorology also needs to be examined [35,75].

During the de-escalation phases, NO and NO_2_ followed a similar pattern, gradually increasing concentrations with the reduction of restrictions, but keeping far away from the reference scenario. PM_10_ and O_3_ showed a more complex trend (see Figure 7), not easily related to the progressive lifting of COVID-19 limitations. Finally, with the Second State of Alarm, there were some falls, but generally of smaller magnitudes than in March (−48% NO_2_).

Nonetheless, as our findings have so far only been derived from the raw data, we will discuss them in detail if, after disentangling meteorological and trend influences from the observed data, there are substantial modifications.

### 3.2. Estimated Changes

The estimated results obtained using the BRT models of the “deweather” package are presented and discussed in this section. Figure 6 shows an example of the application to NO_2_ at the Santander Centro site of the meteorological normalisation described in Section 2.3.1 and the emission pattern trends normalisation explained in Section 2.3.2. As can be seen in the example, the model removed the noise in the graph, which probably corresponded to daily concentration peaks resulting from extreme values of some meteorological predictors; therefore, this procedure makes it easier to clearly observe the response to COVID-19 lockdown on pollutant concentrations. Thus, the percentage change was the gap between the darkest lines (see Figure 6). Again, this comparison was obtained for each pollutant at each site, and the results were summarised and compared with the observed ones in the box plot shown above in Figure 7 (series in green).

From this figure, pollutant concentration changes over the calendar year both from deweathered/detrended and raw data generally demonstrated a similar pattern, although in some cases the magnitude of the change varied slightly, mainly for O_3_. Furthermore, in general, the interquartile range of the concentrations underwent a minimisation after the correction because of the subtraction of meteorological variability.

Pre-lockdown could be considered as a validation step in which the model performance could be tested. In an ideal scenario in which the model is able to fully decouple the influences of weather and emission trends, the percentage change (2020 vs. 2013–2019) in pre-lockdown would be virtually null. As can be witnessed in Figure 7, the model improved the gap in the pre-lockdown period, except for NO_2_. The best results were found for the cases of PM_10_ (+20%) and O_3_ (−10%), where the average variation moved closer to zero. Nevertheless, the starting point was slightly far from the ideal situation. Petetin et al. [53] also obtained a moderate positive bias before lockdown, in Madrid and Barcelona, using a machine learning model. A possible reason for these biases is the lack of some additional predictors that can improve the performance of the BRT model and then the normalised results. Among these, the atmospheric stability and/or planetary boundary layer height can be a key factor, having a significant influence on pollutant dispersion and accumulation [40]. Mor et al. [85] discussed that the increase in air temperature associated with the onset of summer season had a direct impact on the stability of the atmosphere, decreasing it and thus increasing the mixing height of pollutants, which lead to the increment of the vertical mixing ratio in the troposphere. Despite the relevant importance of this variable, it was also not introduced in the model, as no data were available from the sources used. Apart from these considerations, there are many other potential explanations for anomalies in the data, from failures in the measurement equipment to episodic cases of garbage burning [31], or construction work in the surroundings of a monitoring site. In addition, the contributions of African dust outbreaks to the levels of PM_10_ were not included among the model predictors.

Furthermore, the model did not fully eliminate the NO outliers (see Figure 7) during de-escalation phases, but it did reduce their magnitude, so it is reasonable to think that they were the result of unusual episodes not only related to meteorological conditions, taking into account that they were all recorded at the same station (Tetuán, urban background).

With respect to the lockdown period, the normalisation procedure did not produce large changes in the calculation of the relative variations for NO, NO_2_ and PM_10_ at most of the sites, as observed in Figure 7. However, the relative change in O_3_ is lower than the observed change, becoming positive values at some sites. In contrast, during the second lockdown, O_3_ relative changes were larger (more negative) than those observed, probably due to the opposite behaviour of NO (smaller decrease in NO levels than observed).

### 3.3. Analysis of the Traffic Sites

The impacts of road vehicle reductions during the lockdown were more dramatic in the close proximity of roads in comparison with more distant urban background locations [86], so variations at traffic sites were individually analysed using raw and normalised data.

**Figure 8:**
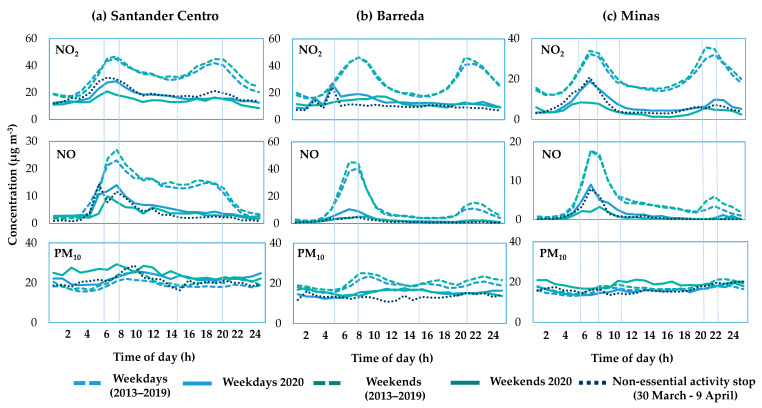
Daily pollutant patterns at traffic sites (**a**) Santander Centro, (**b**) Barreda, (**c**) Minas, during the 2020 lockdown and the equivalent period in the baseline (2013–2019).

To visualise the impacts of traffic restrictions on air pollution, Figure 8 depicts the daily patterns of NO, NO_2_ and PM_10_ at roadsides during 2020 lockdown (from 14 March to 1 May) and the equivalent period of the previous seven years (2013–2019). In addition, weekdays, weekends and the non-essential activity shutdown period in Spain (from 30 March to 9 April) were considered separately, as it is well known that weekly patterns have also a relevant importance in emissions [87]. NO and NO_2_ levels in the baseline scenario (dashed lines) showed a daily profile characterised by two peaks that are related to rush hours [49], around 8 a.m. and 8 p.m., hours in which arrivals and departures in work environments, educational centres, shopping centres, etc., take place. During rush hours, NO_2_ reached the higher levels, of around 50 µg/m^3^, and NO concentrations were over 15–40 µg/m^3^ in the morning. By comparison, the NO second peak was smaller (5–15 µg/m^3^). Looking at Figure 8 it can be clearly confirmed that the lockdown had a major impact on traffic and thus on the levels of traffic-sourced pollutants (solid lines). The typical 8 a.m. peak diminished markedly as NO_2_ and NO did not exceed 30 and 10 µg/m^3^, respectively, and the afternoon peak almost completely disappeared. Regarding the PM_10_ levels, the daily pattern was not as clear, but a drop during lockdown was also observed at some sites, whereas at others there was even a little increase. The differences between working days and weekends were not as remarkable and the cessation of non-essential activity either meant big differences in comparison with the rest of lockdown period.

Globally, when traffic stations were analysed separately, they exhibited a raw reduction in NO_2_ during lockdown with a median value of −56%, which moved to −45% after the correction (see Table 2). These reductions in NO_2_ concentrations at traffic sites, where road traffic is virtually the only source of this pollutant, were only slightly lower than those found for the region on average, suggesting that most of the total reduction found in the region is due to road traffic, with the contribution from industry and other sources being much smaller. For NO and PM_10_, the correction meant less changes, from a reduction of −73 to −70% and from −13 to −18%, respectively.

In relation to the pre-lockdown, the so-called “validation phase”, the median of the estimated values at traffic sites was closer to zero than considering the total sites, reaching −2% for NO_2_, −5% for NO and +14% for PM_10._ These percentages may indicate that the model performed better for the traffic stations, as there is only one main source of pollution there, which can be more easily described, despite not including a predictor variable that accounts for direct vehicle emissions.

Table 2 also shows that the situation after lockdown varied moderately, and it is difficult to state general trends, but overall pollutant levels at traffic sites remained lower than the baseline before and after the normalisation procedure.

## 4. Conclusions

The COVID-19 pandemic and the unprecedented measures adopted during this emergency situation produced side effects on air quality levels due to the large reduction in anthropogenic emissions. This study analysed these effects on air pollutant levels using raw and normalised data series (2013–2020) from a Northern Spanish region (Cantabria), by means of the “deweather” R package, which was used to decouple meteorological and patterns in emission trends from the observed data.

The main outcomes revealed that lockdown restrictions resulted in large reductions in nitrogen oxides, −65% for NO_2_ and −57% for NO; and moderate decreases in PM_10_ (−5%) and O_3_ (−10%). The trend observed for NO_x_ was consistent with strong traffic reductions, as stated in the recent literature. However, the outcomes for PM_10_ and O_3_ were highly variables from study to study, highlighting their complex behaviour and making it difficult to observe general trends. By comparison, the normalised results (“deweathered” and “detrended”) showed relative variations for the studied pollutants in line with observations, exhibiting a general reduction in variability. In addition, the pre-lockdown period revealed anomalous lower observed levels than the reference period for NO_2_ and O_3_ (−23%) and higher levels for NO and PM_10_ (+10 and +34%, respectively); these gaps improved after data normalisation, except for NO_2_, but still were far from zero, evidencing the lack of some additional model predictors.

The results demonstrated air quality improvement, mostly related to traffic reductions, so it is imperative to take measures to boost the transition to sustainable mobility by means of the creation of traffic reduction policies (e.g., implementation of Low Emission Zones (LEZs) and electrification of the vehicle fleet). As a longer-term strategy, authorities are advised to develop an effective integrated policy to reduce emissions from other sectors by replacing fossil fuels with sustainable alternatives, and by raising public concern on environmental issues, as the health of the population is at stake.

## Figures and Tables

**Table 1:** Names, codes, type and monitored parameters of the air quality monitoring sites used in the study.

Site	Code	Type	NO_2_	NO	PM_10_	O_3_	Meteorology *
Castro Urdiales	es1578a	Urban background	✔	✔	✔	✔	✔
Corrales	es1579a	Industrial	✔	✔	✔	✔	✔
Guarnizo	es1576a	Industrial	✔	✔	✔	✔	✔
Cros	es1577a	Industrial	✔	✔	✔	✔	✘
Reinosa	es1530a	Urban background	✔	✔	✔	✔	✔
Santander Centro	es1580a	Traffic	✔	✔	✔	✘	✘
Tetuán	es1529a	Urban background	✔	✔	✔	✔	✘
Zapatón	es1038a	Urban background	✔	✔	✔	✔	✘
Barreda	es1037a	Traffic	✔	✔	✔	✘	✘
Minas	es1039a	Traffic	✔	✔	✔	✘	✘
Los Tojos	es1531a	Rural	✔	✔	✔	✔	✔

* Meteorological data for sites that do not have their own meteorological station were obtained from the Spanish Meteorological Agency station at the Santander–Parayas airport.

**Table 2:** Change (%) in pollutant concentrations at traffic sites for each period in the region under study, using raw and normalised data (dwdt).

Site/Pollutant		Pre-Lockdown		Lockdown		Phase 0		Phase 1		Phase 2		Phase 3		New Normality		2nd State of Alarm	
		Raw	dwdt	Raw	dwdt	Raw	dwdt	Raw	dwdt	Raw	dwdt	Raw	dwdt	Raw	dwdt	Raw	dwdt
**Santander**	**NO_2_**	+3.2	−2.3	−48.4	−42.8	−60.4	−55.8	−45.8	−51.5	−23.7	−32.4	−35.8	−37.7	−50.4	−49.6	−66.8	−62.1
	**NO**	+13.6	−18.8	−59.1	−62.1	−43.4	−48.1	−38.4	−46.3	−73.9	−63.8	−58.0	−56.8	−44.8	−40.9	−64.3	−62.3
	**PM_10_**	+52.1	+38.6	−2.7	−2.2	+4.0	−5.3	+11.5	+5.8	+44.6	+21.9	−15.1	−2.3	+4.3	−0.3	−10.9	−1.6
**Barreda**	**NO_2_**	+9.9	+10.1	−56.1	−45.3	−50.4	−58.8	−58.7	−58.5	−43.1	−52.1	−44.3	−49.1	−46.8	−49.5	−49.5	−48.4
	**NO**	+29.2	+1.5	−77.0	−69.6	−51.0	−58.3	−42.5	−52.3	−28.1	−42.0	−18.9	−36.7	−27.3	−27.5	−47.1	−38.7
	**PM_10_**	−2.9	+3.0	−31.2	−33.4	−30.7	−34.9	−24.7	−30.9	−16.9	−27.5	−49.4	−41.0	−29.9	−30.5	−33.1	−28.0
**Minas**	**NO_2_**	−14.9	−20.3	−68.8	−69.8	−71.9	−67.7	−45.8	−55.5	−44.9	−52.1	−51.5	−51.8	−42.5	−44.7	−48.0	−30.4
	**NO**	+11.7	−4.6	−72.9	−72.6	−76.9	−67.4	−54.1	−59.2	−55.5	−52.0	−57.0	−48.0	−44.6	−40.8	−62.3	−29.9
	**PM_10_**	+20.6	+14.2	−12.8	−18.2	−21.7	−28.0	−15.2	−19.8	−0.7	−12.9	−47.0	−38.2	−35.4	−35.6	−31.7	−27.0
**Median**	**NO_2_**	+3.2	−2.3	−56.1	−45.3	−60.4	−58.8	−45.8	−55.5	−43.1	−52.1	−44.3	−49.1	−46.8	−49.5	−49.5	−48.4
	**NO**	+13.6	−4.6	−72.9	−69.6	−51.0	−58.3	−42.5	−52.3	−55.5	−52.0	−57.0	−48.0	−44.6	−40.8	−62.3	−38.7
	**PM_10_**	+20.6	+14.2	−12.8	−18.2	−21.7	−28.0	−15.2	−19.8	−0.7	−12.9	−47.0	−38.2	−29.9	−30.5	−31.7	−27.0
